# Variation in Antimicrobial Resistance in Sporadic and Outbreak-related *Salmonella enterica* Serovar Typhimurium

**DOI:** 10.3201/eid1501.080853

**Published:** 2009-01

**Authors:** Eva Møller Nielsen, Mia Torpdahl, Steen Ethelberg, Anette M. Hammerum

**Affiliations:** Statens Serum Institut, Copenhagen, Denmark

**Keywords:** Antimicrobial agents, resistance, Salmonella Typhimurium, Salmonella genomic island 1, pulsed-field gel electrophoresis, multiple-locus variable analysis, dispatch

## Abstract

The prevalence of different antimicrobial resistance profiles and variants of the *Salmonella* genomic island 1 (SGI1) was reported for *Salmonella* *enterica* serovar Typhimurium DT104 strains isolated from patients in Denmark. Variation in antimicrobial resistance and corresponding changes of SGI1 were shown among isolates from a foodborne outbreak.

Phenotypic and genotypic typing methods are important for surveillance and outbreak detection of *Salmonella* infections. The stability of the markers assessed by the typing method helps identify clusters and the source of outbreaks (*1*). The phenotypic tests, including antimicrobial-drug resistance profiles and phage typing, are usually considered very stable and therefore often used for surveillance of *S. enterica* serovar Typhimurium. To obtain the discrimination needed for outbreak investigations, high-discriminatory molecular methods like pulsed-field gel electrophoresis (PFGE) and multiple-locus variable number of tandem repeats analysis (MLVA) are favorable. However, band shifts in PFGE and changes in number of repeat units in MLVA can occur for epidemiologically linked isolates. DT104 is the most prevalent phage type in Denmark, accounting for 21% of the *S.* *enterica* ser. Typhimurium cases in 2006. Most DT104 isolates are resistant to at least ampicillin (A), chloramphenicol (C), streptomycin (S), sulfonamides (Su), and tetracycline (T) (ACSSuT). The 5 resistance genes are found in a multidrug resistance (MDR) region that is located on the chromosome in a region termed *Salmonella* genomic island 1 (SGI1) (*2*). Recently, SGI1 has also been found in other *Salmonella* serovars (*3*), and several variants of the SGI1 MDR regions have been described (*4*).

## The Study

We report on the prevalence of different antimicrobial-drug resistance profiles among Danish *Salmonella* ser. Typhimurium DT104 strains from human infections. Furthermore, we show results indicating that a DT104 outbreak strain lost resistance genes by shifting to the SGI1-B variant during the outbreak. Consequently, the outbreak included isolates with both the ACSSuT and the ASu phenotype.

During 2003–2006, 307 isolates of *Salmonella* ser*.* Typhimurium DT104 and 16 DT104b were obtained from persons with clinical disease in Denmark. Several outbreaks and clusters of DT104 were detected; some have previously been reported (*5**–**8*). The 323 isolates were characterized by MLVA (97% of isolates), PFGE (78%), and susceptibility testing (100%). Susceptibility to a standard panel of antimicrobial agents (www.danmap.org) was determined by microbroth dilution and interpreted according to Clinical and Laboratory Standards Institute (CLSI) guidelines (*9*), except for ciprofloxacin (>0.125 µg/mL was used as breakpoint). The 5-locus MLVA described by Lindstedt et al. (*10*) and PFGE, according to the PulseNet protocol (*11*), were performed and analyzed as previously described (*5*). To obtain a cluster-independent data set, we reduced the 323 isolates to 146 unique strains on the basis of the MLVA and resistance profile (i.e., with different MLVA and/or resistance profile) (Table 1).

**Table 1:** Antimicrobial resistance profile of 323 clinical *Salmonella* *enterica* serovar Typhimurium DT104 isolates, Denmark, 2003–2006*

Phenotype	SGI1 genotype†	Unique types, no. strains (%)	All isolates, no. (%)
ACSSuT‡	SGI1§	122 (84)	251 (78)
ACSSuTTm¶	SGI1-A	5 (3)	9 (3)
ASu	SGI1-B	4 (3)	24 (7)
SSu	SGI1-C	3 (2)	11 (3)
ASSuTTm	SGI1-C	1 (0.7)	2 (0.6)
ASSu	SGI1-B	1 (0.7)	2 (0.6)
ACST#	SGI1	1 (0.7)	1 (0.3)
SSuT	Not present	1 (0.7)	3 (0.9)
Sensitive	Not present	8 (6)	20 (6)
Total		146 (100)	323 (100)

*One isolate per multilocus variable number of tandem repeats analysis (MLVA) and antimicrobial resistance profile is represented in the data set of 146 strains with unique types. A, ampicillin; C, chloramphenicol; S, streptomycin; Su, sulfonamides; T, tetracycline; Tm, trimethoprim. †SGI, *Salmonella* genomic island. Genotype determined by the SGI1-related PCR assays described in the text (1 isolate per “unique type”); see Table 2 for selected details. ‡Additional antimicrobial-drug resistances: nalidixic acid and ciprofloxacin (22 strains), amoxicillin/clavulanic acid (3 strains), gentamicin (1 strain). §Only a small subset of 10 ACSSuT strains was tested by PCR for the presence of SGI1 genes. ¶Additional antimicrobial-drug resistances: nalidixic acid, ciprofloxacin, and gentamicin (1 strain). #Additional antimicrobial-drug resistances: nalidixic acid and ciprofloxacin.

Strains with phenotypes lacking resistance to >1 of the antimicrobial agents (ACSSuT) typical for SGI1 were further investigated for the presence of SGI1 variants. PCR was performed for detection of the SGI1 left and right junctions by using primers U7-L12, LJ-R1, 104-RJ, and C9-L2 (*2*); detection of the integron conserved segments (5′-CS and 3′-CS) was done by using primers L1 and R1 (*3*); and detection of the antimicrobial resistance genes *aad*A2*,* *qac/sul1*, *flo*R, *tet*(G), and *bla*P1 was performed by using the primers described by Boyd et al. (*12*).

Most DT104 and DT104b isolates displayed the phenotype ACSSuT (Table 1), which is compatible with the possession of SGI1; 122 (84%) of the 146 strains were resistant to ACSSuT with or without additional antimicrobial drug resistance that is regarded as independent of the SGI1 (e.g., flouroquinolones). Three percent of the strains were additionally resistant to trimethoprim (Tm) and therefore likely to possess the SGI1-A variant, and 6% of the strains were fully sensitive (all of these were DT104). The remaining strains had a variety of resistance profiles, including profiles that comply with the descriptions for variants SGI1-B and SGI1-C (Table 1).

Forty isolates were related to a restaurant foodborne outbreak in 2005. This outbreak was shown to be caused by raw beef, served as *carpaccio* at a restaurant in a limited period of 4 weeks (*6*). The isolates displayed variation with all applied typing methods: phage typing, MLVA, PFGE, and resistance profile; 33 isolates were typical MDR DT104 (ACSSuT) with 1 PFGE and MLVA profile. One isolate differed in phage type (nontypeable) and had a variant of the PFGE profile that differed by 1 band. Three isolates had different MLVA types (variation in 1 of the 5 loci) (*6*). Three other isolates were resistant to only ampicillin and sulfonamide. Detection of SGI1-related genes and regions by PCR showed that the ACSSuT isolates were positive in all PCRs as expected for SGI1 (Table 2), whereas the 3 ASu isolates were positive for the left/right junction, but only 1 integron and *pse-*1 and *qac/sul1,* in accordance with the phenotype and the SGI1-B variant, were positive (*3**,**12*). The most likely mechanism for the creation of variants of SGI1 is considered to be homologue recombination between identical DNA segments (*12*). SGI1-B can thus be generated from SGI1 by a single crossover at *intl*1. Recombination could have taken place in the intestine of the infected animal, during processing or storage of the beef, or during infection of the patients. It is less likely that the outbreak was caused by multiple unrelated strains of DT104 because both the PFGE and MLVA types, including the variants, were distinctive and unique to this outbreak.

**Table 2:** Characterization of *Salmonella* genomic island 1 in isolates with atypical antimicrobial-drug phenotype and isolates likely to be related (possible clusters or outbreaks)*

Outbreak (no. isolates)	MLVA profile	PFGE pattern	Phenotype	SGI1 variant	Left/right junction	*intI1* L1-R1 1.0/1.2 kb	*pse-1*	*florR*	*aadA2*	*qac/ sul1*	*tet*(G)
*Carpaccio* outbreak 1 (33)	253	205	ACSSuT	SGI1	+/+	+/+	+	+	+	+	+
*Carpaccio* outbreak 2 (3)	253	205	ASu	SGI1-B	+/+	−/+	+	−	−	+	−
Pork outbreak 2003–2004 (18)	133	14	ASu	SGI1-B	+/+	−/+	+	−	−	+	−
Jun 2003 “cluster” (2)	437	14	ASu	SGI1-B	+/+	−/+	+	−	−	+	−
Jun 2003 (1)	437	14	ACSSuT	SGI1	+/+	+/+	+	+	+	+	+
Oct 2005–Jan 2006 (8)	443	14	SSu	SGI1-C	+/+	+/−	−	−	+	+	−
Jan 2006 (1)	443	14	ASSuTTm†	SGI1-C	+/+	+/−	−	−	+	+	−
Dec 2003 (1)	309	14	ASSu‡	SGI1-B	+/+	−/+	+	−	−	+	−
Sep 2006 (1)	270	nd	ACST	(SGI1)	+/+	+/+	+	+	+	+	+
Aug 2003 (3)	681	14	SSuT§	−	−	−	−	−	−	−	−
2003–2006 (8)	Various	Various	Sensitive	−	−	−	−	−	−	−	−

*MLVA, multilocus variable number of tandem repeats analysis; PFGE, pulsed-field gel electrophoresis. †Other genes detected by PCR: *bla*, *str*AB, and *tet*(B). ‡Other genes detected by PCR: *str*AB and *sul*2. §Other genes detected by PCR: *str*AB *sul*2, and *te*t(A).

In addition to the 3 isolates related to the *carpaccio* outbreak, 21 human *Salmonella* DT104 isolates in 2003–2006 had the phenotype ASu. All of these had the same PFGE profile (worldwide, the typical DT104 PFGE profile) (*13**,**14*) but 3 different MLVA profiles (represented by 18 isolates, 2 isolates, and 1 isolate each). The cluster of 18 isolates had the phenotype ASu and the MLVA profile 133 (Table 2), and most of these isolates were likely part of an outbreak related to pork from Denmark (December 2003–March 2004). Another cluster of 3 isolates was received in the laboratory within a 3-day period in June 2003 from patients living in the same region. These isolates belonged to a MLVA profile that has not been detected since. The phenotype of 2 of these isolates was ASu, and 1 was ACSSuT (Table 2). No outbreak investigation was carried out at that time, and we can only speculate that this is a similar occurrence of shift in antimicrobial-drug resistance during an outbreak. A cluster of 9 isolates with MLVA 443 occurred late in 2005 and in January 2006; most patients lived in a specific geographic area. Eight of these isolates had the phenotype SSu (equivalent to SGI1-C), and 1 isolate, which appeared late in the cluster, was ASSuTTm. PCR confirmed the presence in all 9 MLVA 443 isolates of the left and right junction, the typical SGI1 genes conferring resistance to SSu, and 1 cassette of class 1 integrons (lacking the *pse*1-associated cassette) (Table 2). The ASSuTTm-isolate was additionally positive for the non–SGI1-related genes *bla*, *str*AB, and *tet*B. In this cluster, it seems likely that the variation in phenotype was caused not by variation in SGI1 but rather by acquisition of antimicrobial-drug resistance genes independent of SGI1 (e.g., on plasmids).

## Conclusions

Most of the *Salmonella* DT104 isolates from patients in Denmark had the typical ACSSuT phenotype; however, a small fraction (6%) of DT104 isolates were fully sensitive and correspondingly negative for all SGI1-related genes and regions tested for by PCR. Approximately 10% of the isolates had a different phenotype, and most of these had an antimicrobial-drug resistance profile corresponding to SGI1-A, -B, or –C, which could be verified by PCR assays mapping parts of SGI1. A number of clusters and outbreaks occurred in the study period, and shifts in antimicrobial-drug resistance phenotype and genotype seem to have taken place in several outbreaks. These shifts were most clearly seen in the *carpaccio* outbreak (6), in which all evidence pointed at a single source outbreak, though the applied typing methods showed some variation among isolates, including a shift from SGI1 to SGI1-B in 3 of 40 isolates. Two other clusters of isolates showed variation in antimicrobial-drug resistance. We therefore conclude that variability of both genotypic and phenotypic characteristics can be expected during a foodborne outbreak; this variability should be taken into consideration when defining outbreak-related cases.
